# Expanding repeats, expanding impact: Somatic instability in myotonic dystrophy type 1

**DOI:** 10.1177/22143602261432779

**Published:** 2026-03-13

**Authors:** Thomas D Hoekman, Nehaa KP Ponraj, Diana Shabshai, Sarah A Cumming, Darren G Monckton, Derick G Wansink

**Affiliations:** 1 Department of Medical BioSciences, Radboud University Medical Center, 6034 Radboud Institute for Medical Innovation, Nijmegen, The Netherlands; 2 School of Molecular Biosciences, College of Medical, Veterinary and Life Sciences, 223860 University of Glasgow, Glasgow, UK

**Keywords:** disease heterogeneity, disease progression, disease severity, DNA repair, mismatch repair, myotonic dystrophy, triplet repeat, repeat expansion, somatic instability

## Abstract

Myotonic dystrophy type 1 (DM1) is a multisystemic neuromuscular disorder caused by a CTG_(n)_ repeat expansion in the 3′-untranslated region of the *DMPK* gene. The repeat tract becomes unstable when exceeding approximately 35 to 50 CTG triplets, expanding both intergenerationally and throughout a patient's lifetime. Somatic instability is age-dependent, tissue-specific, and expansion-biased, with higher levels of expansion correlating with increased disease severity and faster progression. In blood, the estimated progenitor allele length (ePAL) serves as a predictive biomarker for age of onset, while in muscle, the modal repeat length is associated with the degree of muscle impairment. Somatic expansion is driven by DNA mismatch repair (MMR) proteins such as MSH3, and variation in MMR genes have emerged as modifiers of somatic instability and contributors to phenotypic variability. In this review, we evaluate techniques for quantifying repeat dynamics, highlight findings for patient-derived tissues, and discuss insights from animal and cellular models. Together, these advances contribute to a nuanced understanding of DM1 pathogenesis and offer future strategies for disease monitoring and intervention.

## Introduction

Myotonic dystrophy type 1 (DM1; OMIM #160900) is the most common muscular dystrophy in adults, with a prevalence estimated at 1:2100 based on genetic screening.^1^ This autosomal dominant, progressive neuromuscular disorder is considered one of the most heterogeneous conditions caused by a single mutation. This variability is reflected in the age of onset, rate of progression and wide spectrum of symptoms. Organs and tissues commonly affected include skeletal muscle, heart, brain and gastrointestinal tract, with symptoms that can vary considerably between patients, even within the same family.^2^ Clinical presentations are categorised into four overlapping subtypes, ranging from the mild late-onset form to the adult and juvenile subtype, with the most severe being congenital DM1 (CDM) with onset at birth.^3–5^

**Figure 1.**
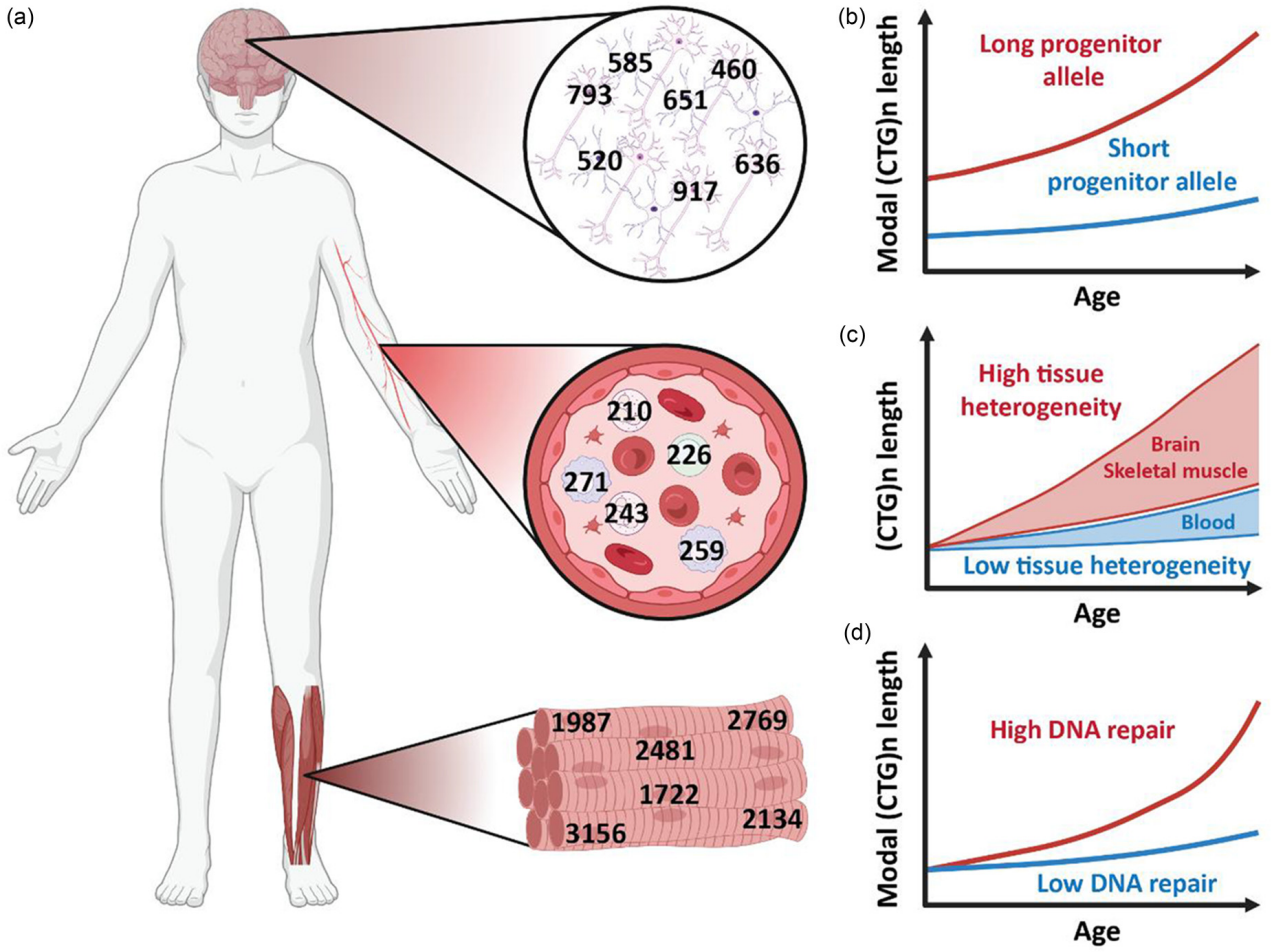
Effects of progenitor allele length, tissue, cell type, and genetic background on CTG repeat-length dynamics. (a) Repeat-length heterogeneity across brain, blood, and skeletal muscle from the same individual, with additional variability observed between cell types within one tissue (e.g., neurones versus astrocytes). (b) Age-related increase in modal repeat length for individuals with short and long inherited allele length, illustrating repeat length-dependent progressive instability over time. (c) Distribution of CTG repeat lengths within different tissues from the same individual, highlighting tissue-specific mosaicism and instability patterns. (d) Influence of DNA repair activity on modal repeat length changes with age, demonstrating that reduced levels of DNA repair, due to tissue-specific differences, genetic variants, or experimental knockdown, lead to reduced instability. Created in BioRender. Hoekman, T.D. (2026) https://BioRender.com/u2j8io4.

DM1 patients inherit an expanded (CTG)_n_ repeat in the 3’-untranslated region of the *dystrophia myotonica* protein kinase (*DMPK*) gene.^6^ The repeat length is polymorphic in the general population, typically ranging between 5 and <35 triplets. Repeats between ∼35 and ∼50 CTGs are generally not associated with symptoms and classified as premutations, whereas in affected individuals, the inherited repeat can range from greater than 50 to well over 2000 triplets. Once the repeat exceeds approximately 35 triplets, it becomes unstable and predominantly expands in both the germline and in somatic tissues,^7–9^ with instability being more pronounced in full-mutation alleles exceeding 50 triplets (Figure 1). Germline instability drives genetic anticipation, with successive generations typically inheriting longer expanded alleles (i.e., the so-called progenitor alleles) that correlate with increased disease severity and earlier age of onset. The repeat is also somatically unstable, with repeat lengths often considerably larger in tissues like skeletal muscle and brain compared to blood.^10,11^ Similar patterns of tissue-specific somatic instability are observed in other repeat expansion disorders, such as Huntington disease,^12,13^ demonstrating a broader mechanism of repeat-length dynamics across diseases.

The primary pathogenic mechanism of DM1 involves RNA-mediated toxicity. Expanded (CUG)_n_ repeats in *DMPK* transcripts form abnormal 3D structures that sequester members of the muscleblind-like (MBNL) family.^14,15^ MBNL proteins are key regulators of developmentally programmed alternative splicing, and reduced functional availability in DM1 results in aberrant processing of various pre-mRNA targets.^16^ A clear repeat-length dependence has been observed for DM1-related mis-splicing.^17^ However, repeat length and age only partially account for disease severity and clinical heterogeneity,^18^ suggesting that additional molecular mechanisms must contribute to pathology. It has been reported that patients whose repeats expand more rapidly than expected tend to exhibit more severe symptoms.^19,20^ Additionally, correlations between aberrant splicing and repeat lengths were strongest when considering the longest disease alleles,^21^ further underscoring the role of somatic expansion in amplifying molecular pathomechanisms and driving disease severity. Interestingly, a distinct subset of patients carry expanded (CTG)_n_ repeat alleles that contain interruptions, generally associated with reduced somatic instability, a milder clinical presentation, and diminished anticipation.^22^ Moreover, increased DNA methylation in the regions flanking the expanded allele is linked with reduced somatic instability, indicating a potential role for epigenetic modifications in repeat stabilisation.^23^

At this point in time, the treatment of DM1 primarily revolves around symptom management and prevention of secondary complications, as a rationally designed disease-specific treatment is not yet available.^24^ Therapeutic development efforts are promising and largely focussing on mitigating RNA toxicity, particularly by targeting the expanded CUG repeat and its downstream effects on splicing regulation.^17,25–28^ However, while these approaches address the primary downstream pathogenic mechanism, their efficacy in splicing correction appears to diminish with increasing repeat lengths,^17^ suggesting ongoing somatic expansion may limit therapeutic responsiveness. This highlights the need to better understand somatic instability, not only as a contributor to DM1 pathogenesis but also as a target for therapeutic intervention. Moreover, linking cell-type specific repeat lengths and instability to clinical symptoms may improve the ability to predict disease progression and stratify patients for tailored therapies. In this review, we evaluate current methodologies for measuring repeat length and dynamics, explore somatic instability in patients and across different tissues, examine the impact of repeat interruptions, discuss insights from animal and cellular models, and address emerging therapeutic strategies targeting somatic instability.

## Evolution of repeat profiling in DM1

Accurate characterisation of the length of expanded repeats is essential, not only for fundamental research into disease mechanisms and therapeutic strategies, but also for clinical diagnosis and patient counselling. Ideally, techniques used for repeat analysis are able to determine a broad range of repeat lengths, quantify somatic mosaicism, and resolve sequence composition, including interruptions and epigenetic modifications. Some of the more commonly used methods are discussed below and summarised in Table 1.

**Table 1.** Commonly used repeat profiling techniques.

Method	Input DNA per sample	Repeat length range covered (in triplets)	Repeat length heterogeneity information	Sequence information (e.g., interruptions)	Epigenetic information (e.g., methylation)
PCR	<100 ng	<150	Smear	No (Sanger sequencing required)	No
Southern Blotting – genomic DNA	≥3 µg ^29,30^, high quality DNA	No limit	Smear	No	No
Southern Blotting – long range PCR	1 ng–100 ng ^31,32^	<1200	Smear	No	No
Triplet Repeat Primed-PCR	50 ng–1 µg ^33,34^	<200 (>200: presence only)	Very little	Presence of interruptions close to the 3’ and 5’ end of the repeat ^35^	No
Small Pool-PCR	3–600 pg ^32,36^	<1200	Yes	Not directly, but can be coupled with *Aci*I digestion to detect most variants ^37,38^	No
Optical Genome Mapping	750 ng, HMW DNA ^21,39^	>>150	Yes	No	No
Short-Read WG Sequencing	2 µg ^40^	<∼40 (>40: indirect estimation ^41^)	Very little for premutations; no information on larger alleles	Yes for premutations; limited information on larger alleles	No
Long-Read Amplicon Sequencing	5–250 ng ^22,42,43^	<1200	Yes	Yes	No
Amplification-Free Long-Read WG Sequencing	1–5 µg, HMW DNA ^44,45^	No limit (in theory)	Very little	Yes	Yes
Amplification-Free Targeted Long-Read Sequencing	1–5 µg, HMW DNA ^46^	No limit (in theory)	Yes	Yes	Yes

WG = whole genome; HMW = high molecular weight.

### Traditional and current diagnostic techniques

The initial step in genetic testing for DM1 involves assessing whether both alleles are within the non-expanded repeat range (≤35 triplets). PCR across the repeat region followed by fragment length analysis can typically detect repeats between 5 and approximately 150 CTGs, covering the lower range of repeat expansions. However, when only one allele is detected, it remains uncertain whether the individual is homozygous for the non-expanded allele or carries a large expansion that remained undetectable. Southern blot hybridisation of restriction-digested genomic blood DNA or long range-PCR products has traditionally been used to resolve this uncertainty.^29–32^ Southern blot hybridisation of genomic DNA is essentially not restricted by the length of the repeat expansion and can reliably characterise even the largest expansions. However, it requires a substantial amount of high quality DNA (∼3 µg), and often yields weak signals against a high, off-target background.^29,30^ Long range-PCR combined with Southern blot hybridisation provides a slightly more practical alternative, requiring less input material (∼15 ng) and tolerating lower DNA quality.^31^ However, even when optimised for long and GC-rich templates, PCR-based approaches often fail to amplify very large expansions of over a thousand triplets.^32^ In any case, both techniques are time-consuming, and rely on input DNA representing thousands of cells, causing the somatically expanded alleles to appear as a broad smear. These methods allow estimation of the modal allele length in the distribution but are not capable of high-resolution quantification of repeat length mosaicism.

Although Southern blot hybridisation of restriction digested DNA is still occasionally used to solve difficult cases, triplet-repeat primed PCR (TP-PCR) has become the preferred method for genetically diagnosing DM1, due to its faster workflow, high sensitivity and specificity, and reduced DNA requirements.^33,34,47^ Unlike classical PCR approaches, TP-PCR uses a fluorescently labelled primer flanking the repeat, together with a repeat primer amplifying from multiple locations within the repeat.^33^ This method generates PCR products that differ in length by three base pairs (i.e., triplets), visualised as a continuous ladder by fragment length analysis. A ladder extending beyond 50 CTG triplets indicates the presence of a disease-causing expansion. Although TP-PCR is effective as a diagnostic tool for detecting expanded alleles, the result is effectively binary and only indicates the mere presence or absence of an expansion, without providing solid information on repeat length and heterogeneity. Repeat interruptions, like CAG, CCG or CGG triplets, may be detected as distinct gaps in the ladder since the repeat primer is unable to bind at the interruption. Notably, these interruptions can also prevent the binding of the repeat primer and lead to false negative results, especially if TP-PCR is performed in only one direction.^35^

### Small pool PCR enables the quantification of somatic mosaicism

**Figure 2.**
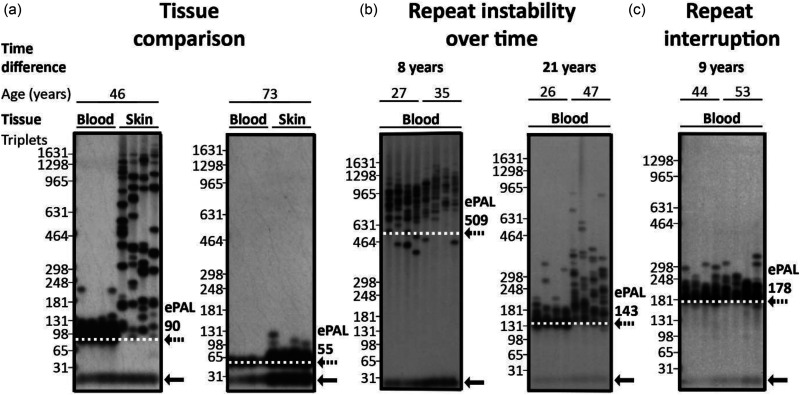
Small Pool-PCR autoradiographs illustrating repeat length heterogeneity and instability. (a) Comparison of repeat length distributions in blood-derived leukocyte and skin-derived fibroblast DNA from individual patients (left panel: age 46; ePAL 90, right panel: age 73; ePAL 55). Note that the amount, quality, or amplification efficiency of the template DNA was lower for blood in the 73-year-old donor, potentially underestimating the degree of mosaicism in this sample. (b) Longitudinal blood DNA samples from individual patients (left panel: 8-year interval; ePAL 509, right panel: 21-year interval; ePAL 143). (c) Longitudinal blood DNA samples from an individual patient with a repeat interruption (9-year interval; ePAL 178). The dotted line and arrow represents the estimated progenitor allele (ePAL) while the solid arrow represents the non-disease associated allele.

Small pool (SP)-PCR is a powerful technique to resolve somatic instability in repeat expansion disorders, addressing limitations of conventional methods like Southern blotting of restriction digested genomic DNA and TP-PCR. To reveal underlying mosaicism often masked by bulk DNA analysis, SP-PCR uses very small amounts of input DNA such that the products of single genomic equivalents can be detected, allowing the qualitative and quantitative detection of individual alleles as distinct signals on an autoradiograph.^32,36^ Repeat length distributions in blood DNA often show a sharp lower boundary. This boundary is conserved between tissues in younger individuals or those inheriting relatively small expansions and provides the best estimate for the progenitor allele length (ePAL, Figure 2) i.e., the number of repeat units inherited from the affected parent.^48^ Estimating the PAL accurately becomes more challenging with age and increasing somatic instability (Figure 2(b)), representing a significant limitation in our ability to fully understand the contribution of inherited repeat length to disease biology in DM1. Although no significant differences in progenitor allele length were observed between SP-PCR and Southern blotting of LR-PCR products from the same sample, SP-PCR yielded slightly longer progenitor alleles.^32^ This finding likely reflects reduced PCR slippage in SP-PCR due to fewer amplification cycles, which better preserves original allele length, as slippage tends to shorten PCR products.^32^ SP-PCR offers single-molecule resolution to study repeat length distribution, even though its detection limit is similar to LR-PCR. Unable to efficiently amplify repeat expansions beyond ∼1200 triplets, SP-PCR is not suitable for the detection of repeat lengths in samples from extreme CDM cases, or longitudinal monitoring of very large somatically expanded alleles in most tissues other than blood.

### Structural profiling of repeat expansions

Addressing the limitations of PCR-based approaches, optical genome mapping (OGM) offers an amplification-free alternative that enables the detection of even very large structural variants and repeat tract lengths.^21,39^ In this technique, single ultra-high molecular weight DNA molecules are fluorescently labelled at specific CTTAAG motifs. When a repeat expansion is present, the physical distance between labels surrounding the repeat increases. By comparing the resulting label pattern to a reference genome, deviations in label spacing can be estimated.^49^ OGM offers estimations of large repeat expansions, with no defined upper detection limit. However, non-expanded alleles already show significant variability in length estimates, with standard deviations of ±100 to 200 CTG units.^21,39^ This variability effectively sets a lower limit for reliable detection, making OGM appropriate only for alleles substantially larger than this threshold. Additionally, similar to SP-PCR, OGM lacks the sequence level context necessary to identify interruptions, an important feature for counselling on disease progression.

### Next generation sequencing in repeat characterisation

To overcome the lack of sequence-level resolution while still enabling quantification of somatic instability, sequencing-based approaches can potentially enable detailed characterisation of repeats. Short-read whole genome sequencing (WGS) can be used to genotype non-expanded and premutation alleles,^40^ but cannot be used to accurately size pathogenic expansions, as the repeat region exceeds the read length. Nonetheless, tools such as ExpansionHunter can be used to support diagnostic workflows by reliably detecting the presence of expanded alleles and estimating repeat length through indirect analysis of short-read WGS data. However, it often underestimates the length of larger expansions, cannot resolve somatic mosaicism, and may provide only limited insight into the presence or absence of repeat interruptions.^40,41^

In contrast, long-read amplicon sequencing utilising either Pacific Biosciences (PacBio) HiFi or Oxford nanopore sequencing (ONT) can allow for accurate detection of repeat interruptions and quantification of repeat lengths in the premutation range,^42^ and up to approximately 1000 CTGs,^22,43^ beyond which PCR-based approaches typically fail to reliably amplify larger expansions.

Amplification-free long-read WGS strategies using PacBio or ONT sequencing enable direct analysis of high molecular weight DNA, allowing accurate sizing of pathogenic expanded alleles, identification of interruptions and assessment of methylation patterns.^44,45^ However, low read depth (typically 20 to 30x), high cost and throughput requirements have thus far limited routine clinical implementation, the reliable estimation of progenitor allele length and/or the quantification of somatic mosaicism. Recently, amplification-free targeted long-read sequencing techniques such as ONT's adaptive sampling and PacBio's PureTarget Panel have emerged. These approaches enrich specific loci using bioinformatic (ONT) or Cas9-based strategies (ONT and PacBio),^46,50,51^ resulting in deeper coverage (typically 10 to 20x for adaptive sampling and 100 to 200x for Cas9-based strategies) and improved cost efficiency. By capturing all relevant features, i.e., repeat length, repeat heterogeneity, and sequence composition, within one single assay, amplification-free targeted long-read sequencing represents a promising and feasible diagnostic solution for DM1 repeat length analysis.

## Somatic instability in patients with DM1

Early molecular studies using patient material revealed that the CTG repeat in the *DMPK* gene appeared as a heterogeneous smear on Southern blots of restriction digested genomic DNA, indicating intra-individual variability and somatic instability.^6,9,52–54^ Subsequent studies confirmed that this instability is tissue-specific, as CTG repeat length varies significantly between organs from the same patient (Figures 1 and 2(a), Table 2).

**Table 2.** Tissue-specific repeat length distribution.

Tissue (cells)	Repeat distribution	Quantitative comparison	Characterisation technique(s)
Blood (leukocytes)	Low heterogeneity; mosaic “smear”	Typically smaller than in muscle; modal alleles ∼200–500 triplets in adult patients^6,11,48^	SB, SP-PCR, long-read sequencing
Skeletal muscle	Largest expansions; strong somatic mosaicism	Often ∼1000–5000 triplets; median ∼2000 in cohort studies^11,55,56^	SB, SP-PCR
Brain (frontal cortex/striatum)	Substantial expansions; region-dependent tissue heterogeneity evident	Approaching muscle levels; often >1000 triplets in post-mortem cohorts; evident heterogeneity^11,57^	SB, SP-PCR, OGM
Brain (cerebellum)	Minimal expansions	Consistently smaller than frontal cortex/striatum, similar to blood; cortical cerebellum shorter than cerebellar white matter^57^	SB, SP-PCR
Skin (fibroblasts)	Intermediate expansions; mosaicism present	Larger repeats compared to blood but shorter than muscle; frequently >1000 triplets ^55,58^	SB, SP-PCR
Heart (myocardium)	Large expansions	Frequently >1000 triplets in post-mortem analyses^11^	SB
Saliva (buccal epithelial cells)	Little repeat heterogeneity	Moderately higher than in blood ^59^	SB, SP-PCR
Liver	Intermediate profile; mosaic “smear”	Larger repeats compared to blood but shorter than muscle ^11,48^	SB
Embryonic/Foetal tissue	CDM cases show large expansions	Commonly >1000–2000 triplets; uniform expansion across multiple foetal tissues^60,61^	SB

SB = Southern blotting; SP-PCR = Small pool-PCR; OGM = Optical Genome Mapping.

### Tissue-specific repeat heterogeneity

#### Blood

As the most readily available tissue, blood remains the most practical and widely studied sample for molecular analysis in DM1.^62^ Longitudinal studies of blood DNA have shown that, with age, both the most frequent (i.e., modal) repeat length and the maximum allele length increase over time.^19^ However, the degree of repeat length heterogeneity varies considerably between individuals. In some, the lower boundary of the smear observed in SP-PCR, representing the ePAL in blood samples, remains relatively stable, whereas in older individuals and/or those inheriting larger alleles it gradually shifts upward^7,19,63^ (Figure 2(b)). It was found that the estimated inherited allele length in blood DNA is a stronger predictor of age of onset than the modal repeat length, which is confounded by age-dependent somatic expansion and does not reliably reflect the progenitor allele. Both longer progenitor alleles and high rates of somatic instability are strongly associated with earlier age of onset, earlier development of symptoms, and increased disease severity.^7,19,38,64–67^

In adults, comparing different individuals at a single time point in cross-sectional studies can be confounded by repeat length-dependent ascertainment bias. Individuals inheriting larger expanded alleles have an earlier disease onset and are sampled young. Conversely, individuals inheriting smaller expansions have a later disease onset and are typically sampled at older ages. As somatic expansion is more strongly driven by inherited repeat length, cross-sectional studies can appear to suggest that somatic expansion goes down with age. Longitudinal studies with repeated samples from the same individuals however, convincingly demonstrate the age-dependent accumulation of somatic expansions and an association between somatic expansion dynamics and disease progression.^19^ Although blood is a practical sample for monitoring individual repeat dynamics,^19,66^ repeat length distributions in blood DNA often fail to capture the severity of tissue-specific involvement, as expansions are generally much larger in affected tissues such as muscle.^11,55^

#### Skeletal muscle

Among the various tissues examined, skeletal muscle has received particular attention due to its central role in DM1 pathology. Early studies first demonstrated pronounced repeat heterogeneity in this tissue,^11^ showing a level of expansion far exceeding that observed in blood. In adults inheriting an expanded repeat of several hundred triplets, expansions in skeletal muscle DNA are typically much longer and can reach 3000 to 5000 CTGs.^10,11,58,68^ As modal repeat length in muscle is highly age-dependent, it does not predict age of onset.^69^ However, modal repeat length in muscle is associated with muscle impairment at a given time.^55^ Studied in only a limited number of patients and muscle types, repeat expansions were found to be similar across different skeletal muscles from the same patients, specifically quadriceps and biceps.^11^ Additionally, post-mortem analysis of a single patient revealed that repeat length and distribution in psoas, gastrocnemius and diaphragm were also comparable to those in quadriceps and biceps.^11^ Moreover, the rate of somatic expansion in muscle is correlated to age of onset, with individuals whose skeletal muscle repeats expanded faster than expected (after correcting for age at sampling and ePAL), the age of disease onset was lower than expected.^20^

#### Brain

Neuronal tissues in DM1 also undergo somatic repeat expansion with marked regional features. A post-mortem study showed that expansions in an adult patients brain were larger than in peripheral blood.^11^ The smallest increase in repeat length was observed in the cerebellum compared to other tissues.^57^ This has also been documented for other repeat expansion disorders such as Huntington disease, spinocerebellar ataxia type 1 and dentatorubral-pallidoluysian atrophy.^70^ In the cerebellum, CTG repeat length was shortest in the cortical regions, moderate in the dentate nucleus and longest in the white matter and middle cerebellar peduncle.^57^ Optical mapping of expanded CTG repeats revealed extreme mosaicism in the frontal cortex, with all examined patients showing repeat lengths over 1000 CTGs, and more than 80% exceeding 4400. These repeat lengths were also correlated with splicing abnormalities, underscoring their functional relevance.^21^ Repeat instability and CpG methylation levels varied across cell type in the central nervous system, where cortical neurones showed longer and more unstable repeats compared to white matter glial cells.^71^ In contrast, spinal motor neurones exhibited shorter repeats and less instability.^71^ However, allele length did not obviously correlate with regional neuropathology.^72^

#### Skin

Cultured fibroblasts from the skin of patients with DM1 exhibited less pronounced repeat expansion compared to skeletal muscle,^55,58,73^ consistent with slower repeat accumulation in fibroblasts. Nonetheless, recent multi-tissue studies showed that allele lengths in skin often exceed 2000 triplets, and correlate with muscle expansions. In fact, the degree of somatic expansion in skin serves as a predictor for expansion in muscle.^20^ As expected, the length of the estimated progenitor allele from blood was highly predictive of the modal repeat length in both muscle and skin^58^ (Figure 2(a)).

#### Saliva

Saliva is considered a convenient non-invasive source of DNA for DM1 studies. In a small cohort, the modal repeat length was shown to be moderately higher in buccal epithelial cells than in blood leukocytes, with the lower end of the distribution also slightly upward shifted; however, the overall degree of somatic variability in saliva was lower than in blood.^59^ Blood-based ePAL estimates explained a greater proportion of the variability in age of onset than salivary ePAL; thus, saliva can be used as an accessible and non-invasive material, but blood-based measures are preferred for predictive models of age of onset.^59^

#### Other tissues

Although data about the myocardium is scarce, individual post-mortem series point to a tendency of large repeat expansions in the heart.^11^ Liver tissue was characterised by a high degree of mosaicism, with larger alleles compared to blood, but shorter than those in skeletal muscle.^11,65^ The repeat heterogeneity observed in the lung showed even greater variability compared to liver, without a clear correlation with patient age.^48^ Large repeat expansions in the gastrointestinal tract, endocrine organs, blood vessels, and peripheral nerves have been reported in post-mortem cases. However, due to limited sample size, reliable estimates of expansion ranges cannot be provided.^11,74^

Given that very large somatic expansions are observed in nearly all tissues other than blood, buccal cells, and the cerebellum, it seems likely that somatic expansion is a key driver of the widespread pathology observed in DM1. Similarly, individual differences in somatic expansion rates across tissues likely contribute to the individual-specific patterns of organ involvement observed in DM1. However, a direct causal role for somatic expansion in disease progression across most tissues has not been established, highlighting the need for more combined multi-tissue analyses and natural history studies. Such studies would help provide the insights needed to predict disease severity and advance our understanding of the disease.

### Role of repeat interruptions on somatic instability

Following the discovery of the *DMPK* mutation, early sequencing studies in a very small number of cases revealed the expansion to be a pure (CTG)_n_ repeat,^6,75,76^ and this was generally assumed to be true for the vast majority, if not all DM1 cases. However, around 17 years later, systematic analyses revealed the presence of repeat interruptions, mostly CCG, and less frequently CGG, CAG, and CTC, within the repeat tract of expanded alleles.^35,37,77–79^ Repeat interruptions have been identified in approximately 3 to 9% of DM1 individuals, and in some cohorts of adults onset patients in up to 10 to 11%.^46,80–82^ Different types and structural configurations of repeat interruptions, occurring at either end of the expanded repeat tract, including a single interruption and *de novo* interruptions, are generally associated with reduced somatic instability (Figure 2(c)).^66,77,81,83^ To our best knowledge, it is currently unknown whether different interruption sequences exert differential effects on reducing somatic instability. Repeat interruptions also correlate with a later age of onset, compared to expectations based on ePAL alone, with delays ranging from 7 to 23 years in individual cases.^81^ To date, repeat interruptions have not been reported in congenital DM1 cases,^80,84^ consistent with the observation that these interruptions are generally protective, especially for the cognitive, and behavioural manifestations of DM1.^55,85^ The vast majority of non-expanded *DMPK* alleles are also comprised of a pure CTG tract. However, in the general population, a considerable fraction of alleles in the premutation length range contain multiple CCG interruptions.^35,37,42,82^ These alleles have a population frequency of 0.35%,^42^ but they are genetically highly stable and are transmitted to offspring with no risk of expansion.^35,37,42,82^

### Modifiers of somatic instability in humans

To uncover additional genetic modifiers of somatic instability, candidate gene studies have been conducted in DM1 and HD cohorts, and genome-wide association studies (GWAS) have been performed in HD. These studies identified the *MSH3* mismatch repair (MMR) gene as a key genetic modifier, with reduced *MSH3* activity associated with a reduced rate of somatic instability and delayed disease onset in both DM1 and HD.^63^ In HD, this variant also correlated with a significantly slower decline in both motor and functional scores, independent of age at onset.^63,86^ Interestingly, DM1 spinal motor neurones, which exhibit shorter repeat expansions and less somatic instability than cortical neurones and white matter glial cells, also show a trend toward lower expression of MMR genes (*MSH2, MSH3* and *MSH6*) and DNA repair gene *FAN1.*^71^ GWAS have also identified variants in the *MSH2*, *MSH6*, *PMS1*, *MLH1*, *MLH3* and *PMS2* MMR genes, and *FAN1* as modifiers of disease severity and/or somatic expansion in HD.^87^ Transcriptome-wide association studies further delineated *FAN1* as a protective modifier in HD, with high expression linked to delayed onset and slower progression.^88^

## What mouse models tell us about somatic instability

Patient studies have revealed key insights into somatic instability and genetic modifiers, but face several limitations: tissue access is restricted, longitudinal sampling is slow, complicated, and often incomplete, and genetic and environmental variability complicate mechanistic interpretation. To overcome some of these challenges, a variety of mouse models, which allow precise genetic manipulation and controlled experimental conditions, have been utilised. Mice also provide access to multiple tissues and developmental stages, enabling targeted investigation of disease mechanisms across the body.

### Mouse models tailored for CUG repeat-induced RNA toxicity

To model muscle-specific symptoms of DM1, mouse models with high levels of CUG RNA expression in muscle were developed. These include the HSA^LR^ mice, with around 250 triplets in the human skeletal actin gene, and the EpA960 mice, which harbour a construct with 960 triplets organised as concatemers with repeat interruptions. These mice showed DM1-like symptoms such as progressive skeletal muscle wasting and degeneration, cardiac abnormalities and provided insights into the expanded CUG repeats, RNA foci formation, MBNL binding and alternative splicing.^89–95^ Despite their value in studying RNA toxicity, somatic repeat instability was not systematically documented in these mouse models, limiting their utility for studying the dynamics of repeat expansion over time.

### Modelling somatic instability in mice

To understand the variable multisystemic effects of unstable CTG repeats observed in DM1, different mouse lines have been generated over the past three decades. Dmt-D mice were one of the earlier models of transgenic mice with 162 CTG triplets and ∼750 bp of the human DM1 repeat locus as flanking sequence. These mice showed that the repeats were unstable in the germline with sex-specific effects.^96^ These mice also showed dramatic, expansion biased, age-dependent, tissue-specific somatic instability.^97^ Tissues from liver, kidney and striatum had more instability, but unlike in humans, these mice did not show much larger repeat expansions in muscle.

(CTG)_84,_ a knock-in mouse model with 84 CTGs in the endogenous *DMPK* gene was specifically generated to study somatic instability.^98^ These mice exhibited tissue-specific somatic expansion, particularly in liver, kidney, small intestine, and stomach, but showed only limited intergenerational instability, with only a few extra repeat expansions per generation unlike the dramatic and frequent changes observed in humans.

DM55 and DM300 mouse lines were generated around the same time, each carrying a 45 kb fragment of the human DM1 region containing 55 and 300 CTG triplets, respectively.^99,100^ Homozygous DM300 mice showed very mild disease symptoms, more prominent in brain than muscle, correlating with the level of CUG RNA expression in these tissues. The DM300 line exhibited sex-specific intergenerational instability and breeding for >10 years resulted in mice carrying over 1000 CTGs, showing clearer disease symptoms.^97,100–102^ The CTG repeats in these mice were also somatically unstable in an expansion-biased, tissue-specific, and age-dependent manner, similar to what we can observe in DM1 patients.^97,100–102^ These expansions have also been observed to accumulate in non-dividing cells in a replication-independent manner, consistent with findings in DM1 patients.^103^

### Uncovering the role of mismatch repair in somatic expansion

To gain insights about the influence of mismatch repair on repeat instability, the (CTG)_84_, DM300 and Dmt-D mice were crossed with mismatch repair gene deficient mice. *Msh6* deficient mice showed slightly greater somatic instability, whereas the repeat was completely stabilised, somatically and intergenerationally, in *Msh3* deficient mice.^98,104^ Interestingly, in *Msh2*-deficient mice, the instability was shifted to having more contractions than expansions.^105^ Moreover, mice with ATPase-defective *Msh2*, neither contractions nor expansions were observed, highlighting the significant function of the ATPase domain in mediating somatic instability.^106^ *Pms2*-deficient mice showed an increase in large somatic deletions, and a reduced rate of somatic expansion.^107^ HD, Fragile X and other disease model mice have also shown that MMR genes, like *Msh3, Msh2*, *Mlh3,* and the DNA repair gene *Fan1*, modify somatic instability, demonstrating that somatic expansion of an unstable trinucleotide repeat occurs through a shared mechanism involving DNA repair, particularly mismatch repair.^108–112^

In conclusion, DM1 mouse models carrying an expanded (CTG)_n_ exhibit varying degrees of somatic instability, likely influenced by their genetic configuration and repeat length. Although generally less pronounced than in patients, somatic instability in these models is tissue-specific, age-dependent, expansion-biased, and occurs even in non-dividing cells, reflecting key features observed in humans. Importantly, the ability to cross these mice with DNA repair-deficient lines has enabled studies demonstrating that mismatch repair pathways play a central role in driving somatic expansions, underscoring conserved mechanisms shared with other repeat expansion disorders.

## Cellular models for studying somatic instability

Mouse models have provided valuable insights into modifiers of repeat instability, showing patterns similar to those observed in patients. Complementary to this, human cell models offer genetically accurate and controllable systems to study repeat dynamics across diverse cell types.

### Somatic instability in pluripotent stem cell models

Human pluripotent stem cells, including induced pluripotent stem cells (iPSCs) and human embryonic stem cells (hESCs), have become valuable models to investigate somatic instability in DM1. Baseline allele length (BAL), defined as the repeat length measured in the original cell line at an early passage, appears to correlate with the degree of repeat expansion in iPSCs during culturing.^113^ Clones with relatively short alleles (57 CTGs) showed negligible expansion over 16 passages, whereas iPSCs with moderately longer alleles (126 CTGs) began to show a measurable increase in repeat length, gaining about one triplet per passage.^113^ Notably, repeats in the range between 773 to 998 triplets displayed higher instability, with expansions of approximately 20 CTGs per passage.^113,114^

Similarly, in hESCs, the rate of somatic instability has been shown to increase with CTG repeat length. In cultures starting with a BAL of 250 triplets, the average expansion rate was approximately three triplets per passage as the repeat length increases to 370.^115^ Between 370 and 465 triplets, the average expansion rate rose to around five triplets per passage, indicating a clear length-dependent acceleration of instability even within these lower repeat ranges.^115^ In hESCs with a BAL of 470 triplets, both contractions and expansions were observed in earlier passages.^116^ Expansion rates averaged around eight triplets per passage within the 470 to 700 repeat length range, where expanded alleles became predominant over time. Instability and variability further increased as culture progressed, where repeat lengths could reach up to 2100 triplets at passage 120. Multiple regression analyses confirmed a significant positive association between passage number and repeat length, underscoring the progressive instability in hESC.^115,116^ It is important to realise that, without information on culture duration or cell division rates, passage number offers only a limited perspective on repeat dynamics. Nevertheless, these findings underscore a distinct, length-dependent increase in repeat instability during extended culturing of stem cells.

### Differentiated cell types reveal modulators of somatic instability

Building on these observations in pluripotent stem cells, parallel analyses have shown that CTG repeat instability diminishes following differentiation. In both iPSCs and hESCs, differentiation into embryoid bodies, neurospheres and other disease-relevant lineages such as cardiomyocytes, neurones and myocytes lead to stabilisation of the CTG repeat length,^113–115^ often within six weeks of differentiation.^113^ Importantly, since the genetic background and repeat length remained constant between pluripotent and differentiated models, these findings point towards the involvement of distinct modifiers influencing repeat instability.

Pluripotent stem cells exhibit high expression of mismatch repair genes. However, upon differentiation, key mismatch repair genes like *MSH2*, *MSH3*, and *MSH6* are downregulated at both transcript and protein levels, correlating with repeat stabilisation.^113–115^ Notably, shRNA-mediated knockdown of *MSH2* significantly reduced repeat expansion rates in pluripotent stem cells. In a stem cell line carrying 600 CTGs, *MSH2* knock-down resulted in repeat stabilisation, concurrent with a gradual loss of methylation upstream of the repeat.^117^ In contrast, the chromatin in somatically stable DM1 cardiomyocytes adopted a closed conformation at the *DMPK* locus and the *SIX5* promoter, unlike the open state seen in controls.^114^ Notably, this raises questions about whether chromatin compaction represents a protective mechanism for repeat instability, contributes to pathogenicity, or is simply a correlative feature without direct functional impact.

Furthermore, the zinc finger protein ZNF850 has emerged as an apparent novel regulator of repeat instability. Its downregulation in a particularly stable iPSC clone, along with RNA interference experiments showing reduced expansion upon knockdown, supports a functional role in promoting instability.^118^ ZNF850 binds directly to the expanded CTG repeat and is located physically near MutSβ components, potentially facilitating repeat expansion by recruiting the MMR machinery to the repeat region.

## Mechanism of somatic instability

Insights from candidate gene studies in humans, mouse studies and cell models have revealed that mismatch repair genes such as *MSH2*, *MSH3*, *MSH6*, *PMS1*, *PMS2*, *MLH1*, *MLH3* are modifiers of somatic instability in DM1 and other repeat expansion disorders.^63,67,86,113–115^^,117–119^ FAN1 is also a well-known modifier, although its contribution to somatic instability has not been specifically demonstrated in DM1 yet. Polymorphism in the human *MSH3* gene has been shown to modify instability in both DM1 and HD, suggesting a common DNA repair mechanism that is responsible for instability.^63,67,120^

The activity of these mismatch repair genes on repeat expansions has been hypothesised to have the undesired effect of generating expansions when they interact with non-B form DNA structures. CTG repeats can mis-align to form stable non-B form slipped strand DNA structures,^121,122^ while repeat interruptions reduce the formation of these abnormal configurations.^123^ Formation and repair of slipped strand DNA structures occur on unwound DNA and are independent of replication.

**Figure 3.**
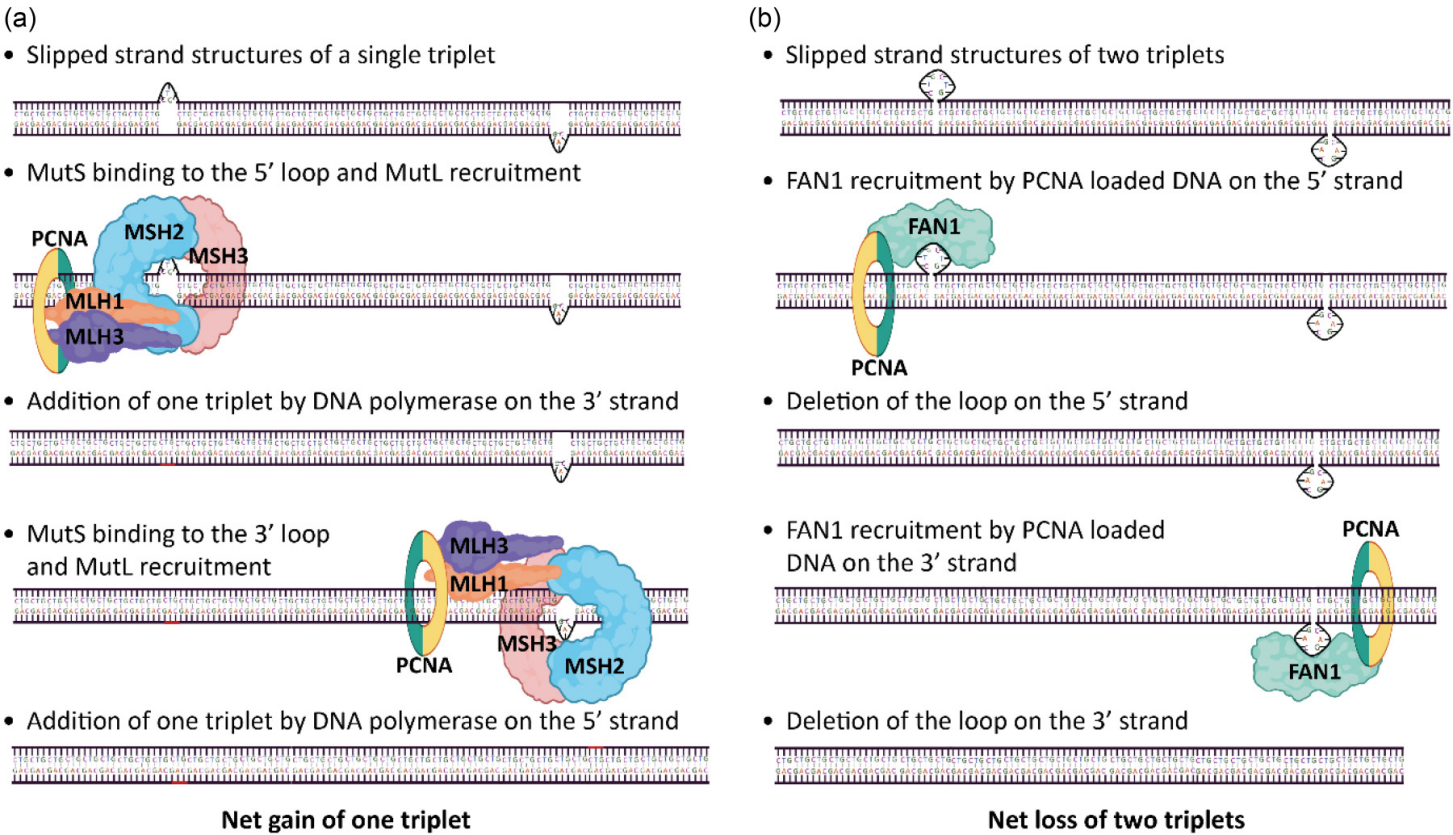
DNA repair pathways and repeat dynamics. (a) MutSβ-mediated repair of repeat extrusions promotes repeat expansion. (b) FAN1-mediated repair leads to contractions. N.B. An important role for *FAN1* in somatic instability has been demonstrated, but not yet specifically for DM1. Created in BioRender. Ponraj, N.K.P.

The mismatch repair MutS complexes detect abnormal DNA structures. The heterodimer MutSα (MSH2/MSH6) recognises base-base mismatches and small loops, whereas MutSβ (MSH2/MSH3) detects larger loops up to 12 nucleotides.^124^ Both MutSα or MutSβ can subsequently recruit MutL complexes. Recruitment of MutLα (PMS2/MLH1) is associated with canonical MMR activity, while recruitment of MutLβ (MLH1/PMS1) and MutLγ (MLH1/MLH3) is linked to repeat expansions, particularly when recruited by MutSβ. The replication sliding clamp PCNA activates the endonuclease activity of MutLγ, introducing a strand break in the strand opposite to the loop. In the context of expanded repeats, DNA polymerase then fills the gap by using the loop as a template, incorporating additional triplets into the locus.^125,126^ In slipped strand structures, this erroneous repair can occur independently on both strands, resulting in a net gain of CTG triplets (Figure 3(a)). Alternatively, when an extrusion of two to three triplets is present, FAN1 nuclease is recruited by PCNA and introduces a nick in the strand containing the extrusion. Repair of this nick leads to the removal of the extrusion^127^ (Figure 3(b)). The size of the extrusion determines which pathway it follows, two to three triplet extrusions can be repaired by FAN1, promoting repeat contraction, while slip-outs of a single triplet, and up to four triplets, are processed by MutSβ, promoting expansion.^124,127^ Importantly, as MSH3 and MSH6 compete for binding to MSH2, MSH6 may exert a protective effect by limiting MutSβ formation.^98^

## DM1 therapies: A prospective shift in focus

Recently, there has been a focus on development of molecular therapies acting on lowering expanded *DMPK* gene expression and/or correcting dysregulated alternative splicing.^17,26,128^ Some of these approaches include transcription inhibition, degradation of expanded CUG RNA, blocking MBNL binding to CUG RNA, upregulating MBNL1, and targeting CELF1 and other molecules.^17,26,128^ Very excitingly, recent clinical trial data indicate that *DMPK* lowering is able to mediate meaningful improvements in the regulation of alternative splicing and in some downstream phenotypes.^129,130^ However, a key challenge for the long term efficacy of therapies targeted downstream of the DNA will be that the repeat tract will be expanding over time making it more challenging to mitigate RNA toxicity.^17^

Gene editing strategies such as CRISPR-Cas9-mediated excision of the expanded repeat or TALEN-induced repeat contractions offer a more direct approach to eliminating the pathogenic allele.^17,73,131,132^ While promising, these methods still face significant challenges related to delivery, efficiency, and safety.

As shown above, mismatch repair-driven somatic instability has been established as a driver of disease progression in DM1, HD and other trinucleotide repeat disorders, making the mismatch repair pathway a desirable target for development of therapies.^133^ Single nucleotide polymorphisms in *MSH3* have been associated with lower rate of somatic instability and reduced disease progression.^63^ In HD models, therapeutic suppression of the mismatch repair pathway, achieved by inhibition or reducing expression of non-cancer associated mismatch repair proteins like MSH3, PMS1, and MLH3 using ASOs and small molecules, has been shown to directly reduce somatic repeat expansion.^134–137^ Upregulation of FAN1 activity is another strategy that is not only focussed at preventing disease progression, but also potentially for symptom reversal.^137^ Companies such as LoQus23 Therapeutics, Harness Therapeutics and Ionis Pharmaceuticals are currently developing drugs that target somatic instability in HD, which can hopefully be extended towards DM1 in the future.^134,138,139^

## Conclusions and outlook

Somatic instability of expanded CTG repeats in *DMPK* is a key driver of DM1 severity and disease progression (Figure 1). Technical advances have transformed repeat profiling, moving from Southern blotting and SP-PCR to amplification-free approaches such as optical genome mapping and long-read sequencing. These methods enable direct visualisation of large expansions, interruptions, and epigenetic modifications, providing unprecedented resolution of repeat dynamics.

Patient studies have revealed that somatic instability in DM1 is highly tissue-specific, with expanded CTG repeats showing variable length and dynamics across organs. In blood, both longer progenitor alleles and higher rates of somatic instability are strongly associated with earlier age of onset and increased disease severity. However, the relatively low levels of heterogeneity observed in blood underestimate the extent of expansion observed in more affected tissues like muscle and brain.

Studies in patients, mice and cellular models have identified MMR genes (*MSH2*, *MSH3*, *MSH6, MLH1, MLH3*, *PMS1*, *PMS2*) and *FAN1* as key modifiers of somatic instability in repeat disorders, acting as either expansion enhancers or suppressors. Instability arises when MMR complexes process slipped-strand DNA, with MutSβ promoting expansion and FAN1 favouring stabilisation.

Targeting mismatch repair to reduce repeat expansion has already shown promise in HD and should now be transferred to DM1. To capture the dynamics and tissue-specificity of somatic instability and assess therapeutic impact, longitudinal studies in clinically relevant tissues such as muscle are essential. Integrating amplification-free targeted long-read sequencing approaches could further enhance the ability to resolve large repeat expansions and assess the contribution of epigenetic modifications. These efforts will be critical for refining our understanding of somatic instability in DM1, linking this to patient symptoms and identifying biomarkers that better reflect disease progression.
